# Factors associated with physicians’ behaviours to prevent needlestick and sharp injuries

**DOI:** 10.1371/journal.pone.0229853

**Published:** 2020-03-16

**Authors:** Fu-Li Chen, Peter Y. Chen, Jeng-Cheng Wu, Ying-Lin Chen, Tao-Hsin Tung, Yu-Wen Lin

**Affiliations:** 1 Department of Public Health, College of Medicine, Fu Jen Catholic University, Xinzhuang, New Taipei City, Taiwan; 2 Department of Psychology, Auburn University, Auburn, AL, United States of America; 3 Department of Urology, Taipei Medical University Hospital, Taipei City, Taiwan; 4 Department of Occupational Medicine, Taipei Medical University Hospital, Taipei City, Taiwan; 5 Department of Medical Research and Education, Cheng Hsin General Hospital, Pai-Tou, Taipei City, Taiwan; University of Sydney, AUSTRALIA

## Abstract

**Objective:**

Needlestick and sharp injuries (NSIs) experienced by physicians have been identified as a major occupational hazard. Blood-borne pathogens resulting from the NSIs experienced by physicians pose severe physical and psychological threats to them, as well as people who are around them. However, there is little research focusing on physicians’ behaviours to prevent NSIs. In the present study, we investigated the roles of safety climate, job demands experienced by physicians, and physicians’ self-efficacy in affecting physicians’ behaviours to prevent NSIs.

**Methods:**

401 physicians from four teaching hospitals in Northern Taiwan were recruited to participate in an anonymous survey. Among them, 189 physicians returned the completed survey with a response rate of 47.1%.

**Results:**

Overall, respondents reported frequently engaging in NSI prevention behaviours. As expected, safety climate in hospitals and physicians’ self-efficacy to prevent NSIs were significantly related to their behaviours to prevent NSIs (r = 0.22 and r = 0.33, respectively). The moderating analysis also revealed that physicians with high self-efficacy tended to engage in NSI prevention behaviours regardless of levels of job demand they experienced. In contrast to our expectation, however, physicians with low self-efficacy engaged in more NSI prevention behaviours when job demands were high than when the demands were low.

**Conclusions:**

Our findings show the important roles safety climate, job demands and self-efficacy play in shaping physicians’ NSI prevention behaviours. Hospitals may consider improving safety climate via strengthening management commitments to NSIs prevention, reducing job demands by training physicians to proactively redesign their own jobs, and increasing physicians’ self-efficacy via well-designed skill-based training.

## Introduction

Needlestick and sharps injuries (NSIs) experienced by health care workers (HCWs) have been identified as a major occupational hazard. It has been estimated that 600,000 to 800,000 NSIs occur each year, with about half of these injuries not being reported [1]. High rates of NSIs have been reported ranging from 14.9% to 69.4% [2] worldwide including North America [3], eastern European countries [4], western European countries and Russia [5], Saudi Arabia [6], and various Asian countries (India, Singapore, Taiwan, Korea, and China) [7]. NSIs have significant adverse impacts on physical and psychological health as well as productivity loss [8]. Furthermore, there are more than 60 communicable blood-borne pathogens, such as human immunodeficiency virus (HIV), hepatitis B virus (HBV), and hepatitis C virus (HCV), resulting from exposure to NSIs [8,9], and about 2.1 million HCWs each year are infected with HIV, HBV and HCV due to the exposure of NSIs [8].

Among HCWs in Taiwan, physicians (4.3%) suffer NSIs more often than nurses (2.7%) based on Taiwan Exposure Prevention Intervention Networks [10]. Thus, it is important to investigate factors that are associated with NSIs among physicians. Past research in the US, China, and Japan has shown that NSIs tend to happen when HCWs experienced high patient load, long work hours, poor safety climate, insufficient or inadequate personal protective equipment (PPE), or failure of complying with standard procedures to handle needles and sharps [1, 11–13]. However, there is little research in Taiwan that specifically focuses on physicians’ behaviours to prevent NSIs, which plays a key role in reduction of NSI incidents.

To address the aforementioned gap, the present study investigates to what extent job demands, self-efficacy of preventing NSIs, and safety climate are associated with physicians’ NSIs prevention behaviours. Job demands, safety self-efficacy, and safety climate have been shown to be related to safety performance [11, 12, 14–16], although their roles in physicians’ NSIs prevention behaviours have not been investigated.

Safety climate is employees’ shared perceptions about the relative importance of safe conduct at work when compared to other priorities such as patient care. It reflects how safety regulations, policies, programmes, and behaviours are practiced, monitored, and valued across levels and units of hospitals. Hence, safety climate, either explicitly or implicitly, informs employees how serious a hospital and its management are about safety practices and safety priorities. Safety climate also serves as a frame of reference and norms to guide employees what to do and what not to do, which would inevitably affect physicians’ perceptions and expectation of what behaviours are valued and monitored [17,18]. Safety climate has been shown to be a significant and important leading indicator of safe practices across industries and occupations. For instance, safety climate has been repeatedly found to predict the increase of safety practices amongst HCWs [19, 20]. Recent meta-analyses also provided consistent evidence that safety climate was positively associated with safety behaviours [21–23].

In sum, a strong safety climate in hospitals sends a clear message that management teams are concerned about work hazards such as NSIs, and would encourage physicians to take all necessary precautions to prevent NSIs. Thus, we hypothesize that safety climate is positively related to physicians’ NSIs prevention behaviours (H_1_).

Job demands have been linked with the reduction of safety behaviours. Sampson et al. reported that job demands such as safety uncertainty (e.g., receiving conflicting instructions from different supervisors) and safety obstacles (e.g., being provided with inadequate resources to perform tasks) were negatively associated with safety behaviours in construction workers [24]. Research focusing on various types of job demands also exhibited similar results across occupations including nurses [25], teachers [26], police officers [27], or project managers [28]. While experiencing high job demands such as high volume of patients, physicians tend to treat patient care as the top priority, and likely have limited cognitive capacity to recognize surrounding needlestick hazards. As a result, job demands likely interfere with physicians’ ability from engaging in safety behaviours to prevent NSIs. Thus, we expect a negative relationship between job demands and physicians’ NSIs prevention behaviours (H_2_).

Even hospitals have strong safety climate that encourages management teams to execute NSIs prevention policy, a high level of job demands would likely attenuate the positive impact of safety climate on physicians’ NSIs prevention behaviours. While facing high demands at work, physicians tend to work faster and spend longer hours treating patients, and at the same time fail to follow safety procedures (e.g., avoid recapping needles, dispose used needles in appropriate sharps disposal containers) to reduce NSIs. Following the above rationale, we hypothesize an interactive effect between safety climate and job demands on physicians’ behaviours to prevent NSIs (H_3_). Specifically, the positive impact of safety climate on physicians’ NSIs prevention behaviours is likely weaken when physicians experience high job demands. In other words, physicians experiencing high job demands would engage NSIs prevention behaviours less often even regardless levels of safety climate in hospitals.

In addition to job demands and safety climate, physicians’ self-efficacy of preventing NSIs may play an important role to prevent NSIs. Self-efficacy is referred to as one’s confidence in their ability to engage a particular behavior or performing a task [29] such as preventing NSIs in the present context. Self-efficacy also reflects one’s belief about his/her “capabilities to mobilize the motivation, cognitive resources and courses of actions needed to meet given situational demands” [30]. Compared to their counterparts, people with high self-efficacy of a particular behavior (e.g., preventing NSIs) may be more resilient and motivated when they encounter obstacles (e.g., high job demands) that restrict the behavior of interest. Past research has shown that self-efficacy was associated with an improvement of job performance [14,31] and motivation [32]. Thus, we expect a positive relationship between self-efficacy of preventing NSIs and physicians’ NSIs prevention behaviours (H_4_).

Furthermore, in line with the definition and past empirical findings, physicians with high self-efficacy would likely be more motivated than those with low self-efficacy to avoid NSIs by frequently engaging in NSIs prevention behaviours, even when they encounter higher job demands. Given that self-efficacy would mobilize resources to complete tasks while facing job demands, we expect that self-efficacy of preventing NSIs would counter the adverse impact of job demands on physicians’ NSIs prevention behaviours (H_5_). In other words, physicians with high self-efficacy would engage NSIs prevention behaviours regardless the level of job demands.

## Methods

### Subjects & procedures

All physicians (401) from four teaching hospitals in Northern Taiwan were recruited to participate in the survey. The number of available physicians of each hospital ranged from 76 to 113. An anonymous self-administered survey was distributed to each physician, and 189 physicians returned the completed survey, with a response rate of 47.1%. Written informed consent was obtained from all participants, and the study protocols was reviewed and approved by the Institutional Review Board at Taiwan Fu-Jen Catholic University (No: C9809).

### Measures

The survey consisted of demographic information (gender, age, education, marital status, affiliated department, and job title) and the variables of interest, which were described below.

#### NSIs prevention behaviours

Two items with five response categories ranging from *never*, *seldom*, *sometimes*, *often*, and *always*, were chosen based on interviews with HCWs [33]. Based on a Monte Carlo simulation, the optimum number of response categories to improve reliability and validity of a scale ranges from four to seven [34]. Respondents were asked, over the last 3 months, how often they had discarded used needles and sharp objects into designated containers, and how often they had looked for sharp objects left by other HCWs.

#### Job demands

Five items of a Chinese version [35] of the Job Content Questionnaire [36], with five response categories ranging from 1 (*strongly disagree)* to 5 (*strongly agree*), were used to measure the job demands physicians experienced. Items were slightly modified to be applicable to the health care context when needed. Sample items are ‘I perform excessive work to care for patients’, ‘I have insufficient time to perform my work tasks’, and ‘My job requires hard work’.

#### Safety climate

A 10-item scale with five response categories, ranging from 1 (*strongly disagree*) to 5 (*strongly agree*), was modified for the health care setting based on the safety climate scales developed by Zohar and Luria [37]. Sample items are ‘Compliments workers who pay special attention to safety’, ‘Considers safety when setting our work speed and schedules’, and ‘Insists we wear our protective equipment even if it is uncomfortable’.

#### Self-efficacy of preventing NSIs

Three items with five response categories, ranging from 1 (*extreme lack of confidence*) to 5 (*extreme confidence*), were developed based on DeJoy’s [38] conceptualization of safety self-efficacy (i.e., beliefs about one’s ability to follow indicated safety measures successfully), and interview results with HCWs [33]. These items are ‘I have the confidence to dispose of needles and sharp objects in proper containers after each use’, ‘I am confident of avoiding NSIs while drawing blood from irritable patients’, and ‘I am confident that I will look out for needles and sharp objects left at work area’.

### Data analysis

Means, standard deviations and correlations among the study variables were first conducted. The latter are used to test Hypotheses 1, 2, and 4. After that, a moderated regression analysis was conducted to test Hypotheses 3 and 5.

## Results

Prior to analysis, we examined if there were mean differences on NSIs among hospitals and demographic variables. No significant mean differences were found. Descriptive statistics, coefficient alphas, and intercorrelations among the studied variables are reported in Table 1. Overall, the full range of scores was observed for each scale, although respondents tended to report high NSI prevention behaviours (mean = 6.64; observed range = 0–8). All coefficient alphas were above 0.80 except for NSI prevention behaviours (α = 0.61). Safety climate and self-efficacy to prevent NSIs were significantly related to NSIs prevention behaviours (*r* = 0.22 and *r* = 0.33, respectively). These results supported H_1_ and H_4_. Contrary to H_2_, job demands appeared to be positively associated with NSIs prevention behaviours, although the relationship was not significantly different from zero.

**Table 1:** Descriptive statistics and correlations among the study variables (N = 189).

	Mean	SD	Possible Range	Observed Range	1	2	3	4
1.NSIs prevention behaviours	6.64	1.42	0–8	0–8	0.61			
2.Job demands	18.61	3.21	5–25	10–25	0.13	0.84		
3.Safety climate	34.88	6.35	10–50	13–50	0.22**	0.03	0.95	
4.Self-efficacy of preventing NSIs	11.72	2.13	3–15	4–15	0.33**	0.11	0.22**	0.88

Coefficient alphas are reported in the diagonal.

*p < 0.05

**p < 0.01.

**Fig 1:**
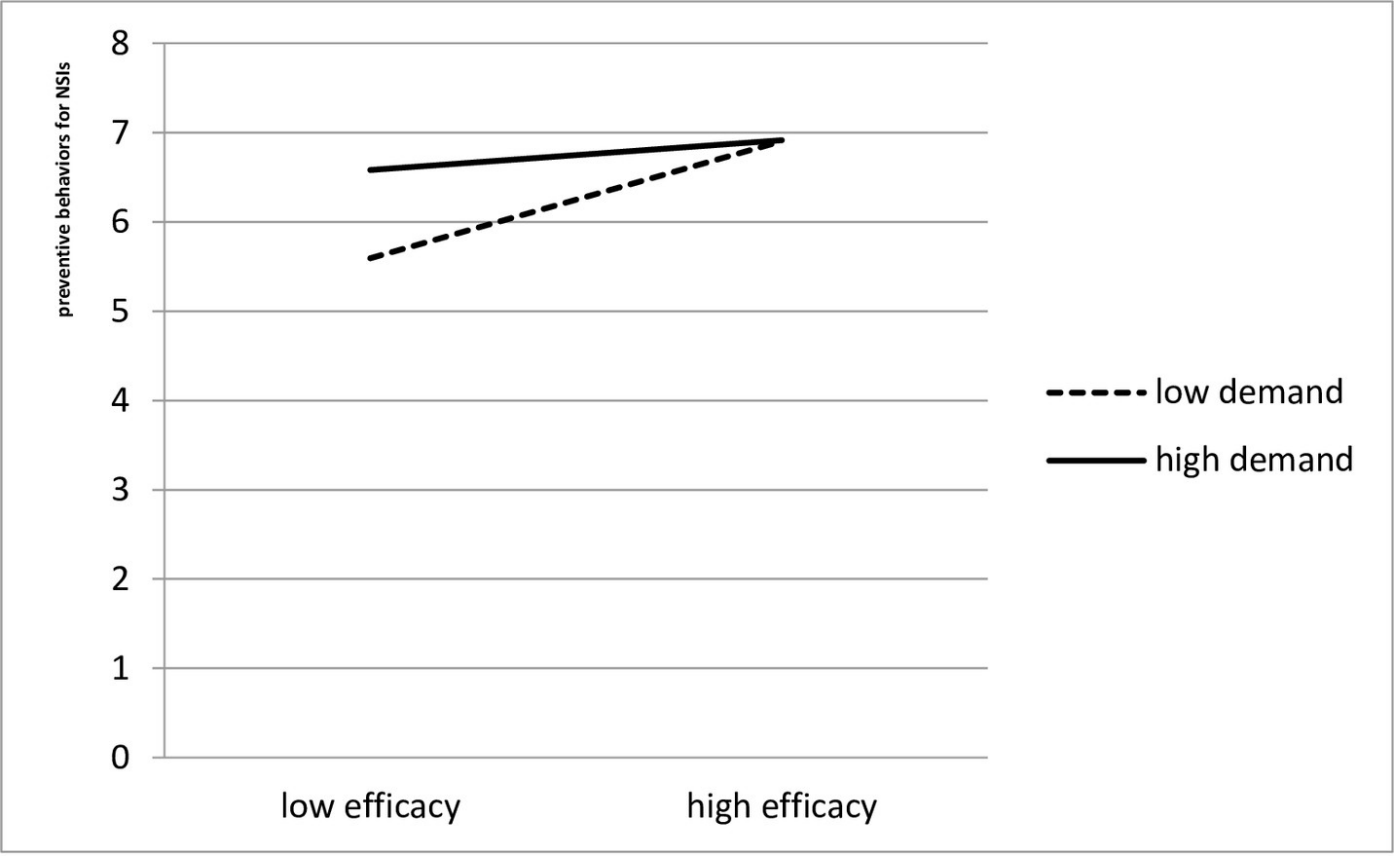
Interaction between self-efficacy to prevent NSIs and job demands in predicting NSIs prevention behaviours.

To test H_3_ and H_5_, as the first step of a moderated regression, ‘NSIs prevention behaviours’ was regressed on job demands, self-efficacy of preventing NSIs, and safety climate. As the second step, two interaction terms, job demands **×** self-efficacy and job demands **×** safety climate, were entered into the regression model. Table 2 shows the results of the moderated regression. Only the interaction between job demands and self-efficacy was significant, which supported H_5_. The pattern of the significant interaction, depicted in Fig 1, revealed that physicians with high self-efficacy tended to engage in NSIs prevention behaviours regardless of the job demands experienced. Counterintuitively, physicians with low self-efficacy engaged in more NSIs prevention behaviours when job demands were high than when they were low.

**Table 2:** Results of moderated regression in predicting NSIs preventing behaviors.

	Step 1			Step 2		
	β	SE	p-value	β	SE	p-value
Intercept	6.65	0.10	0.00	6.67	0.10	0.00
Job demands	0.04	0.03	0.16	0.03	0.03	0.31
Safety climate	0.03	0.05	0.03	0.04	0.02	0.02
Self-Efficacy to prevent NSIs	0.19	0.05	0.00	0.18	0.05	0.00
Demand* Climate				-0.001	0.01	0.92
Demand* Efficacy				-0.04	0.02	0.02
R^2^	0.14			0.18		
Adjusted R^2^	0.13			0.15		
Δ R^2^				0.03		
p-value	0.00			0.03		

## Discussion

Results of the study revealed that safety climate and self-efficacy of preventing NSIs were significantly related to physicians’ NSIs prevention behaviours. In addition, a significant moderating effect on physicians’ NSIs prevention behaviours was found among physicians with high self-efficacy regardless levels of job demands they experienced. However, the results also showed physicians with low self-efficacy engaged in more NSIs prevention behaviours when job demands were high than when they were low. Although it seems intuitive to expect physicians not to fully follow safety practices while experiencing stressful job conditions [39], Leung et al. [40] found that construction workers who suffered physical stress behaved more safely. Lueng et al. suspected that stressful conditions stimulated hormones in workers, which allowed them to cope through fight or flight reactions.

Our results overall indicated that safety climate and self-efficacy may play a significant role to encourage physicians to engage in safety behavers to prevent NSIs. Navon et al. [16] used safety role models, safety training programmes, and rewarding employee safety performance to increase safety self-efficacy. As shown in past research [39,41], an effective training programme should include various skill-based learning to strengthen workers’ beliefs about their abilities to avoid injuries/accidents safety. These recommendations can be applied to hospital settings to enhance physicians’ NSI prevention self-efficacy.

Our findings also suggested that NSI prevention behaviours may be improved by strengthening safety climate in hospitals. Occupational safety research has identified management commitment to safety as one of the most significant factors in improving safety climate [42], which shapes employees’ safety behaviours [43]. Mullen and Kelloway [44] demonstrated that commitment could be strengthened through safety-specific transformational leadership training. Their leadership training also improved leaders’ safety attitudes, intention to promote safety, safety self-efficacy, as well as safety climate perceived by employees. Several studies further demonstrated that safety behaviours and safety climate improved when leaders showed commitment to safety by providing employees with timely feedback pertaining to safety-related behaviours and practices [45–48].

### Limitations

Three limitations should be noted while interpreting results of the present study. First, we used self-report measures to collect data. Results based on self-report measures may be susceptible to common method variance (CMV) [49]. However, given the relatively modest size of the intercorrelations described in Table 1 (mean correlation between studied variables of *r* = .17) and the nature of anonymity, CMV is less likely to have a noticeable impact on the present results [50]. In addition, some respondents may not actually engage in safety behaviors even though they report they do so. To reduce CMV and inaccuracy of self-reports, researchers may consider using mixed methods (e.g., observational methods and self-reports) to assess variables of interest in future research.

The second limitation is the use of cross-sectional design, and no inferences of causality can and should be made. It is important to recognize, however, that cross-sectional designs can provide invaluable information on topics with limited research [51]. Future research should collect data on physicians’ NSIs prevention behaviors and its related antecedents (e.g., safety climate) at multiple time points so it is possible to determine how these antecedents influence physicians’ behaviors to prevent NSIs.

Finally, although the sample is relatively large, it is only from regional teaching hospitals in Northern Taiwan. It is possible that there are certain defining characteristics of these hospitals (e.g., workloads on teaching and patient care) that affect the generalizability of the study’s results. Additional studies that use random sampling or survey multiple hospitals across different regions can help replicate the current findings.

## Conclusions

Past research has shown NSIs often occurred when HCWs recapped needles, transferred a body fluid between containers, or failed to dispose of used needles properly in puncture-resistant sharps container [1]. Although NSIs prevention strategies (e.g., avoiding using needles where safe and effective alternatives are available, using devices with safety features provided by hospitals, planning for safe handling and disposal of needles before using them, etc.) are well documented, NSIs remain a major occupational hazard faced by HWCs every day.

Results of the present study revealed the important roles safety climate and self-efficacy play in shaping NSIs prevention behaviours. Hospitals can improve safety climate by developing strategies to strengthen management commitments to NSIs prevention [48], and provide well-designed skill-based training to increase physicians’ self-efficacy [39,41].

Our study showed that job demands did not appear to be related to NSIs prevention behaviours, and physicians with low self-efficacy tended to engage in more NSIs prevention behaviours while experiencing high job demands. Both counterintuitive results need to be replicated in future research. However, given that the relationship between job demands and NSI incidents were consistently reported in prior research [1, 11–13], hospitals may reduce adverse effects of job demands by training physicians to proactively redesign their own jobs (i.e., job crafting) [52,53].

## Supporting information

S1 Data (XLSX)
